# Sciatica Presentations and Predictors of Poor Outcomes Following Surgical Decompression of Herniated Lumbar Discs: A Review Article

**DOI:** 10.7759/cureus.11605

**Published:** 2020-11-21

**Authors:** Ahmed Aljawadi, Gagan Sethi, Amirul Islam, Mohammed Elmajee, Anand Pillai

**Affiliations:** 1 Trauma and Orthopaedics, Manchester University NHS Foundation Trust, Manchester, GBR; 2 Orthopaedics, Hind Institute of Medical Science, Lucknow, IND; 3 Trauma and Orthopaedics, Wythenshawe Hospital, Manchester, GBR; 4 Spinal Surgery, Royal Orthopaedic Hospital NHS Foundation Trust, Birmingham, GBR

**Keywords:** backache, lumbar disc herniation, radiculopathy, sciatica, disc.

## Abstract

Pain associated with sciatica is one of the most common indications for surgery. The annual rate of discectomy has increased over recent years, with a significant number of patients reporting a poor outcome or symptom recurrence after surgery. This study aims to evaluate the predictors of poor outcome for patients undergoing lumbar discectomy for sciatica. A comprehensive search was conducted to find relevant literature published between 1985 and 2019. All literature with a clear methodology were included. Many factors that affect postoperative recovery after lumbar discectomy have been reported. Some evidence suggests that sociodemographic factors, including female gender, smoking, increased age, low socioeconomic status, and low education level may be associated with less favorable outcomes after surgery. Symptom duration does not appear to be associated with a significant difference in long-term outcomes; however, early surgery (within one year) may result in a faster postoperative recovery with better early results. Furthermore, patients who had discectomy for predominant leg pain had better outcomes compared to those who had the surgery for back pain as the main presentation. There was no evidence to suggest a correlation between the size of the herniated disc and long-term outcomes of sciatica; however, a higher anatomical level of herniation (L1-2, L2-3) was associated with poorer outcomes compared to the lower level of herniation (L3-4, L4-5). A few studies suggested slow postoperative recovery correlates with unemployment and depression. We recommend that the predictors of postoperative outcomes should be taken into consideration when selecting or counseling patients for lumbar disc decompression.

## Introduction and background

Lower-limb nerve root pain caused by lumbar disc herniation is one of the most frequent indications for spinal surgery. The mean annual rate of discectomies in Sweden during the past decade was 24 operations per 1,00,000 [1]. In the US, a marked increase (2.1 per 1000 Medicare enrollees) in the rate of lumbar discectomies was seen in the past decade [2]. A recent literature review showed that about 3% to 43% of patients have a recurrence of back and leg symptoms and a poor outcome following a lumbar decompressive surgery [3]. The high and variable rates of poor outcomes in the literature give pause for thought that is surgery being performed on a lesion which is in fact not the cause of pain. It stresses the need for proper selection of cases and exploration of the reasons for continued pain and poor outcomes after surgery. Therefore, it is important to identify the predictors and factors leading to a poor outcome.

Pain is usually the most important symptom of patients with sciatica, and also the most important factor when selecting patients for surgery. The severity of pain experienced by patients is usually assessed on a visual analogue scale (VAS). An elimination or reduction of pain is essential for surgical success and improved quality of life [1]. In practice, patients with lumbar disc herniation are selected for surgery based on the amount of leg/back pain, neurological symptoms, clinical signs, and correlations with imaging. Some studies have reported that predominant back pain following discectomy for a prolapsed disc is unpredictable [4, 5].

This study aims to evaluate the predictors of poor outcomes after lumbar disc decompression for sciatica. A literature search was performed in the electronic databases of Web of Science, Pubmed, Science Direct, and Google Scholar. In addition, manual searches and cross-referencing of articles were performed to include related reviews, studies, and reference lists. Different search strategies with different combinations of words related to back pain, sciatica, disc herniation were used had been implemented to retrieve the most relevant literature (Table 1).

**Table 1:** Search Strategies Performed Using the Following Search Terms to Identify Relevant Related Articles

No.	Description of Search Strategies
1	((Disc OR Herniaiton OR Decompression OR Minimally Invasive) NOT (Fusion OR Fixation)).
2	“Lumbar Discectomy” AND Predictors
3	1 OR 2
4	((Predictor* AND Outcome*) OR Poor OR Sicaitca, OR Lumbar)
5	Microscopic Discectomy
6	Lumbar Discectomy
7	4 OR 5 OR 6
8	3 AND 7

All studies of predictors of outcomes after lumbar disc herniation surgery published between 1985 and 2019 with a clear description of the methodology were included. Papers discussing the predictors of outcomes after lumbar disc decompression with fusion or stabilisation were excluded. Table 2 summarises the inclusion and exclusion criteria.

**Table 2:** Inclusion and Exclusion Criteria

Inclusion	Exclusion
Sciatica due to lumbar disc herniation	Diagnosis of nerve root compression due to other conditions rather than lumbar disc herniation (e.g: tumor, trauma, or spine degenerative conditions)
Lumbar disc decompression (open or minimally invasive)	Paper described spinal stabilization or fusion
Adult patients (>18 years old)	Other levels of disc herniation rather than lumbar spine
Studies discussing predictors of outcomes after decompression	Conservative treatment

**Figure 1:**
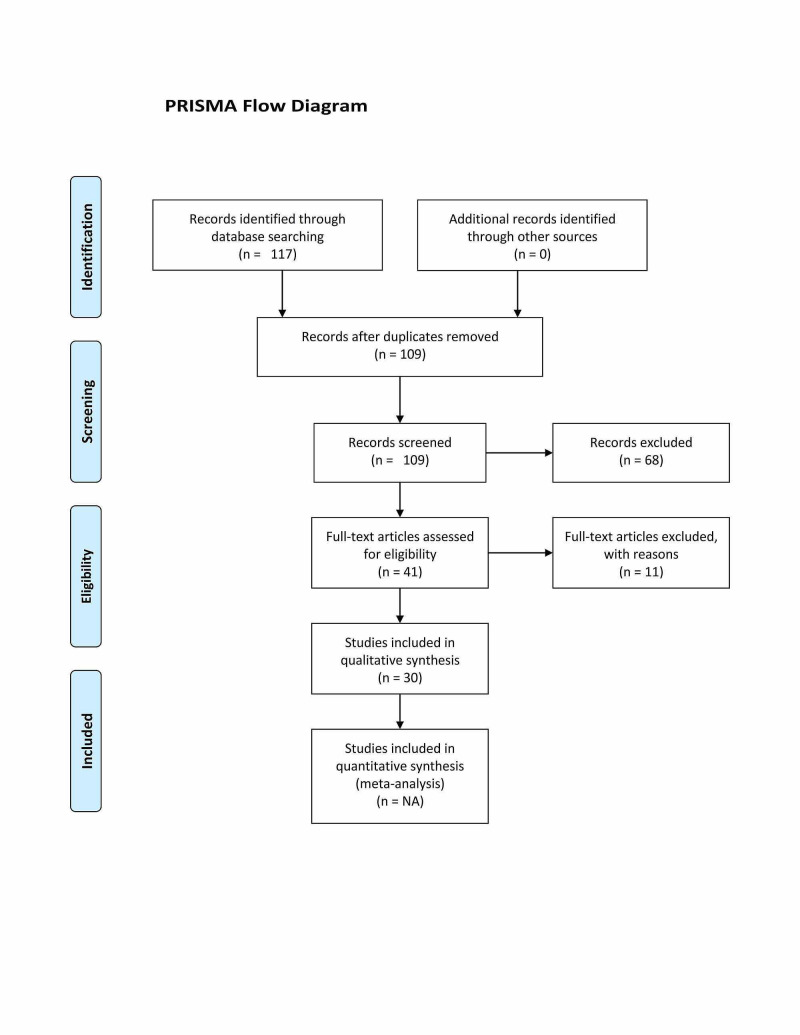
Prisma Flow Chart

Our initial search retrieved 117 papers from the included databases, however, after excluding duplicate records, only 109 papers were subjected for title and abstract review. This review had resulted in the exclusion of another 68 papers. Subsequently, 41 papers were included for full-text review, and only 30 papers out of 41 met the inclusion criteria and were included for this review (Table 3). Articles reviewed following the PRISMA flow chart to ensure adherence to review papers guidelines (Figure 1).

**Table 3:** Studies Included in the Review

No.	Authors	Paper Titles
1	Jansson et al. 2005 [1]	Health-related quality of life in patients before and after surgery for a herniated lumbar disc
2	Peul et al. 2007 [8]	Surgery versus prolonged conservative treatment for sciatica
3	Weber et al. 1993 [10]	The natural course of acute sciatica with nerve root symptoms in a double-blind placebo-controlled trial evaluating the effect of piroxicam
4	Yu et al. 2009 [14]	Imaging study of lumbar intervertebral disc herniation and asymptomatic lumbar intervertebral disc herniation
5	Fraser et al. 1995 [16]	Magnetic resonance imaging findings 10 years after treatment for lumbar disc herniation
6	den Boer et al. 2006 [23]	A systematic review of bio-psychosocial risk factors for an unfavourable outcome after lumbar disc surgery
7	Graver et al. 1999 [24]	Seven-year clinical follow-up after lumbar disc surgery: results and predictors of outcome
8	Hurme and Alaranta 1987 [25]	Factors predicting the result of surgery for lumbar intervertebral disc herniation
9	Häkkinen et al. 2007 [26]	Changes in the total Oswestry Index and its ten items in females and males pre-and post-surgery for lumbar disc herniation: a 1-year follow-up
10	Haugen et al. 2012 [27]	Prognostic factors for non-success in patients with sciatica and disc herniation
11	Kerr et al. 2015 [28]	What are long-term predictors of outcomes for lumbar disc herniation? A randomized and observational study
12	Kara et al. 2005 [29]	Functional results and the risk factors of reoperations after lumbar disc surgery
13	Dewing et al. 2008 [30]	The outcomes of lumbar microdiscectomy in a young, active population: correlation by herniation type and level
14	Soriano et al. 2010 [31]	Predictors of outcome after decompressive lumbar surgery and instrumented posterolateral fusion
15	Lequin et al. 2013 [32]	Surgery versus prolonged conservative treatment for sciatica: 5-year results of a randomised controlled trial
16	Rothoerl et al. 1998 [33]	Are there differences in the symptoms, signs and outcome after lumbar disc surgery in the elderly compared with younger patients?
17	Junge et al.1995 [34]	Predictors of bad and good outcomes of lumbar disc surgery. A prospective clinical study with recommendations for screening to avoid bad outcomes
18	Dionne et al. 2001 [35]	Formal education and back pain: a review
19	Ng and Sell 2004 [36]	Predictive value of the duration of sciatica for lumbar discectomy: a prospective cohort study
20	Postacchini et al. 2002 [37]	Recovery of motor deficits after microdiscectomy for lumbar disc herniation
21	Dasenbrock et al. 2012 [41]	The efficacy of minimally invasive discectomy compared with open discectomy: a meta-analysis of prospective randomized controlled trials
22	Watters and McGirt 2009 [42]	An evidence-based review of the literature on the consequences of conservative versus aggressive discectomy for the treatment of primary disc herniation with radiculopathy
23	Wera et al. 2008 [43]	Reherniation and failure after lumbar discectomy: a comparison of fragment excision alone versus subtotal discectomy
24	McGirt et al. 2009 [44]	A prospective cohort study of close interval computed tomography and magnetic resonance imaging after primary lumbar discectomy: factors associated with recurrent disc herniation and disc height loss
25	Kleinstueck et al. 2011 [45]	The outcome of decompression surgery for lumbar herniated disc is influenced by the level of concomitant preoperative low back pain
26	Lønne et al. 2012 [46]	Recovery of muscle strength after microdiscectomy for lumbar disc herniation: a prospective cohort study with 1-year follow-up
27	Righesso et al. 2012 [47]	Correlation between persistent neurological impairment and clinical outcome after microdiscectomy for treatment of lumbar disc herniation
28	Carragee et al. 2003 [48]	Clinical outcomes after lumbar discectomy for sciatica: the effects of fragment type and annular competence
29	Wittenberg et al. 1998 [49]	The correlation between magnetic resonance imaging and the operative and clinical findings after lumbar microdiscectomy
30	Sanderson et al. 2004 [50]	The unique characteristics of “upper” lumbar disc herniation

Background and pathophysiology of sciatica

Sciatica is a symptom (rather than diagnosis) characterized by radiating pain below the knee into the leg and foot, which is the area supplied by one or more nerve roots from the lumbar or sacral spine. It may be associated with sensory and/or motor deficits, muscle weakness, reflex alteration, or all of them. The most common cause of sciatica is a herniated disc (90%); however, other possibilities include lumbar canal stenosis and foraminal stenosis, or, rarely, tumors or cysts [6-8].

The natural history of lumbar disc herniation is favorable, and spontaneous resolution of symptoms may occur in the majority of cases [9-11]. Severe pain and disability usually resolve over a period of two to four weeks [7], and 60% of patients return to work by the fourth week [10]. At one year, 90% of patients improve significantly [12]. For those who do not respond to conservative measures, surgery provides significant pain relief in the short term, but when it should be done is still controversial [13]. A systemic review comparing the result of surgical and conservative treatment concluded that there was no difference between the outcomes of both groups at one and two years [13]. Knowledge of the natural history of sciatica is essential to determine the time when an intervention should be done. Offering an expensive treatment at a time when the disease has a high likelihood of improvement on its own would be a waste of resources, but if it is done at the correct time and in patients where the disease does not seem to follow a usual pattern, it could have an economic and clinical benefit.

Multiple reasons indicate that pure mechanical compression is not enough to explain the symptoms complex of sciatica [14]. Severe symptoms can be present without evidence of nerve root compression, and the symptom severity does not necessarily correlate with the size of the herniated disc [15]. The outcome of conservative treatment is favorable in the majority of cases, despite the persistence of the herniated disc [7, 16]. Pressure on normal nerve roots is not seen to cause pain; moreover, a discectomy has only a moderate long-term success rate [3]. On the other hand, experimental studies show that discogenic pain can be caused by tears or breaks in the annulus fibrosis [17]. The nucleus pulpous has immunogenic potential and, once out of the confinement of the annulus, incites a strong inflammatory reaction that is the cause of pain [18, 19]. Elevated levels of phosphoLipids A2 (PLA2) and tumor necrosis factor (TNF), “key enzymes in the cascade of inflammation,” have been seen in lumbar disc herniations in symptomatic patients [20, 21]. Therefore, the current belief is that sciatic pain is most likely caused by a complex combination of mechanical compression and biologic processes of inflammation [9, 22].

## Review

Predictors of outcomes of decompressive surgery for lumbar disc herniation

The goal of decompressive surgery is to relieve the pressure on the nerve root thereby decreasing pain. A technically well-performed surgery on the right patient will often result in a good outcome. Therefore, it is important that we identify factors that influence the outcome of surgery as patients who fail to recover are at risk of developing chronic pain syndromes. Over 150 predictor variables have been documented with varying levels of significance [23]. Predictor variables reported in the literature are categorized into socio-demographic, clinical, work-related, and psychological variables. There is no consensus on any of the predictor variables among studies.

Socio-Demographic Variables

Gender: Most studies found female gender to be a risk factor for a poor outcome; whereas one study stated that males were more at risk of a poor outcome [24-27]. A few found no association between gender and outcome [25, 28-31]. Graver et al. in a study of 122 patients observed that females had a significantly higher level of preoperative lower back and leg pain compared to males. At seven years' follow-up, females had worse outcomes and their clinical overall scores (COS) were significantly higher compared to males (F: 223.95; M: 140.37; P = 0.02) [24]. Though this study concluded that outcomes were worse in females, it also stated that of the seven patients who underwent a re-operation, only one was female. Hurme and Alaranta found that though the preoperative indices of pain were the same for both sexes, the indices of activities of daily life were postoperatively worse in females [25].

Age: No consensus exists amongst studies regarding age being a risk factor. A clear line between the young and the elderly has not been drawn. Hurme and Alaranta included 357 patients younger than 55 years and found that at six months' follow-up after surgery the activities of daily living and pain were worse in patients aged 40 and above [25]. Two other studies also found that age over 40 was a predictor of poor outcome whether the patients were operated on or treated conservatively [10, 32]. Rothoerl et al. in his study of 219 patients considered patients above the age of 59 as elderly and found no significant differences between the elderly and the young [33].

Socio-Economic status and education level: Papers have stated that education level correlates with outcome, but the demarcation between the educated and uneducated is not properly defined [34]. Soriano et al. compared results between patients with a primary or elementary education and a secondary or higher education and found that a higher level of education was predictive of a better outcome (better Oswestry Disability Index [ODI] score and less leg pain) [31]. It was seen that better-educated patients had a better psychological mechanism for coping with surgery and interpreting postoperative symptoms positively. It was also observed that the level of education affected the outcome in an indirect way as well, by leading to differences in occupation, socio-economic status, health status, environmental risk factors, differences in access to and utilization of health services, and adaptation to stress [35]. In contrast to the above studies, Dewing et al. did not find education or rank to influence the outcome [30].

Smoking: Many studies found that smokers had a worse clinical outcome, longer recovery period, and reduced quality of life [1, 30, 31, 33]. In a study of 263 patients, pre-operative mean scores of EQ-5D were similar between smokers and non-smokers, but a higher proportion of smokers had not improved at 12 months and had experienced worse scores [1]. However, few studies did not find smoking to be an important risk factor for a poor outcome [24, 29, 36, 37]. Further randomized clinical trials of pre- and postoperative smoking cessation would be required to determine the impact of smoking on the outcome of surgery.

Clinical and Surgical Variables

Time to surgery/duration of pain: How the duration of symptoms before surgery influences functional recovery after lumbar discectomy and what the time point for intervention is beyond which postoperative recovery might be compromised are still unanswered. In the absence of red flags and specific causes (infection, tumors, fractures), there is a general consensus to try conservative treatment for at least six to eight weeks [7]. For patients who fail to show adequate recovery within this time frame and continue to suffer from persistent pain (without serious neurologic deficits), it remains controversial whether they should be operated on early or prolonged conservative treatment should be continued. Pitsika et al. performed a retrospective study on 107 patients divided into four groups based on duration of their sciatica symptoms: Group 1 had symptoms for less than six months, Group 2 had symptoms for 6 - 12 months, Group 3 from 12 to 24 months, and Group 4 were symptomatic for more than two years. Results showed an improvement in pain and function in all groups, without a significant difference between any of the groups; however, the best results were recorded for Group 1 [38]. In the same context, Ng and Sell in a study on 113 patients determined that a greater degree of satisfaction with surgical outcome was observed if patients were operated on within 12 months of onset of sciatica. A duration of more than 12 months statistically correlated with a less favorable outcome as indicated by the ODI score and low back outcome score (Ng and Sell 2004). Peul et al. prospectively studied 283 patients suffering from sciatica for a duration of 6-12 months. Patients were randomly assigned to either have an early surgery performed within two weeks or to receive prolonged conservative treatment with an option of surgery if conservative management failed. Of the 142 patients designated for conservative treatment, 55 (39%) were treated surgically after a mean of 18.7 weeks. The one-year outcomes were similar for patients assigned to early surgery and those assigned to conservative treatment with eventual surgery if needed, but the rate of pain relief and recovery was faster in patients who underwent early surgery [8]. At five years, no difference was seen in pain and disability between the groups [32].

Type of Surgery: Whether minimally invasive surgery results in better overall postoperative outcomes compared to conventional open surgery is still a controversial topic, and the literature failed to show any particular technique to be clearly superior to another [39, 40]. The aim of minimally invasive techniques is to be less destructive and less traumatic to the soft tissue and muscles. Graver et al. found that a wide exposure was significantly associated with a greater postoperative low back pain regardless of whether a full or a partial laminectomy or a single or two-level discectomy was performed [24]. A recent systematic review and meta-analysis by Evaniew et al. did not find minimally invasive procedures to be more effective than open discectomy with respect to function, extremity pain, complications, and reoperation rate. On the other hand, an overall higher rate of nerve root injury, incidental durotomy, and reoperation were reported with minimally invasive surgery compared to open surgery. Another systematic review also suggested that both OD and MID lead to substantial and equivalent long-term improvement with no difference between them [41]. There was no difference in relief of leg pain between the two approaches either in the short-term (two to three months postoperatively, 0.81 points on the VAS) or long-term follow-up (one to two years postoperatively, 2.64 on the VAS) [41]. The advantages of minimally invasive surgical techniques, which include less soft tissue and muscle damage, reduced perioperative blood loss, low infection rate, shorter hospital stay, and faster recovery, would be more relevant for multilevel surgeries and instrumented procedures.

Conservative vs aggressive discectomy: Conservative discectomy results in shorter operative time, quicker return to work, and a decreased incidence of long-term recurrent lower back pain, but also results in an increased incidence of recurrent disc herniation [42]. While subtotal discectomy is more invasive, it has a <1% re-herniation rate [43]. In a study of 108 patients who had conservative discectomy, re-herniation was the cause in 11 (10.2%) patients requiring revision discectomy at a mean of 10.5 months. A larger annular defect and less disc removal were associated with an increased risk of re-herniation while greater volumes of disc removal were associated with accelerated disc height loss [44].

Preoperative level of back and leg pain as a predictor of outcome: Many studies observed that patients with a preponderance of radicular leg pain had better surgical outcomes after decompressive surgery compared to those operated on with back pain as their main complaint [23, 30, 34, 45]. In a study of 308 patients, Kleinstueck et al. found that fewer patients with back pain as their 'main problem' had a good outcome at 12 months (69% good) compared with those who reported leg/buttock pain (84% good) or neurological disturbances (80% good) to be their main problem [45]. Dewing et al. followed 183 young active patients with a mean age of 27 years for three years. They observed that patients with a higher percentage of preoperative back pain did not demonstrate as much postoperative improvement as those with a preponderance of leg pain. The authors recommended lumbar microdiscectomy to be an effective and predictable treatment for radicular leg pain recalcitrant to nonoperative management, but not for isolated lumbar back pain [30]. A recent study on the predictors of outcome of lumbar disc herniation reported that severe baseline back pain was a predictor of poor outcome whether the patients were managed surgically or treated conservatively, but those that were managed conservatively had an even worse outcome; the conclusion was that surgery had a greater treatment effect [28]. More recently, a study of 995 patients by Sethi et al. concluded that patients with lower back pain of 6 or more on VAS are at increased risk of poor outcome following MID [5]. All the above studies have suggested that the greater the preoperative back pain (with sciatica), the worse the outcome. There is evidence to suggest that back pain of 6 or more on VAS could represent the demarcation line for this. 

Neurological impairment: Some studies have reported that neurological symptoms improved after decompressive surgery and the recovery was inversely related to the severity of preoperative paresis [37, 46, 47], but the amount of recovery (from neurological deficits) did not necessarily correlate with or have an effect on outcome scores (e.g., VAS or ODI) [28, 37, 47]. In a study of 91 patients with preoperative paresis due to a disc herniation, 75% had a full recovery with no paresis at 1-year postoperatively [46]. The preoperative duration of the paresis did not influence the rate of full recovery, but the severity of paresis was associated with a fourfold increase in risk for non-recovery [46].

Size and type of herniation: Contained discs were associated with the poorest outcomes, significantly worse than either extruded (P < 0.001) or sequestered (P < 0.001) disc types [30]. The best surgical outcomes and lowest re-herniation rates were reported in discs with small annular tears and large disc fragments, and the worst outcomes with contained herniations and no isolated fragments [48]. In a 10-year follow-up study, a persistent herniated disc was found in 37% of patients, but the presence or absence of persistent disc herniation was not significantly correlated with the symptoms or outcome. These findings indicate that long-term improvement of symptoms may occur with or without resolution of the herniated disc [16]. Other studies have also found that clinical symptoms and improvement following symptomatic lumbar disc herniation do not necessarily correlate with radiological findings [14, 49]. 

Anatomical level of disc herniation: Some studies observed that the clinical presentation and outcomes of upper lumbar disc herniation (L1-L2, L2-L3) were worse compared to those at lower levels (L3-L4, L4-L5, L5-S1). In a study of 69 patients with herniations at L1-2, L2-3, and L3-4 only, it was seen that when compression was present at L1-L2 or L2-L3, 58% had improvement in radicular pain and 53% patients had improvement in back pain. In comparison, when compression was present at L3-L4, 94% of patients had improvement in leg pain and 87% had improvement in back pain [50].

In another study of 197 patients by Dewing et al., disc herniations at the L5-S1 level were associated with significantly better outcomes on VAS leg (not back) score and ODI score than those at the L4-L5 level. They postulated that lumbo-pelvic ligaments provided inherent stability at the L5-S1 level and also that the neural foramen for the S1 nerve root is larger [30].

Occupation and Satisfaction with Work

Various factors related to work were predictive of outcomes for patients undergoing surgery or managed conservatively. In a study by Kerr et al., it was seen that patients who were working at the time of presentation had the greatest relative improvements compared to those who were disabled at the time of presentation [28]. Furthermore, patients who were on restricted duty before the operation, were unemployed or on part-time employment, expressed the desire for early retirement, were receiving workers compensation, had a higher intensity of pain or depression, or had been off work for more than 28 weeks before the operation were at higher risk of not returning to work [30].

Psychological Variables

There is evidence supporting the idea that pain-related fear may be more important in predicting disability than the pain itself. It was seen that patients with a higher level of anxiety, pain coping, or pain catastrophizing, had poorer postoperative outcomes [23, 34]. While patients with a positive estimation of operative results, optimistic preoperative expectations, less preoperative psychological distress, and a good mental component score (emotional health) had a more favorable postoperative outcome (as assessed by leg pain VAS and ODI) [24, 25, 31].

## Conclusions

Spontaneous resolution of symptoms can be expected in up to 90% of patients with sciatica. For those who fail conservative treatment, surgery performed at the right point of time for carefully selected patients can provide significant pain relief. Factors to be considered as predictors of poor postoperative outcomes had been described extensively in the literature, with no consensus regarding any of them. There is no evidence to suggest that a longer duration of preoperative symptoms has a significant impact on the long-term results; however, earlier surgery (within one year) was associated with better early postoperative outcomes and a faster recovery. The literature has also suggested that predominant preoperative back pain, as opposed to leg pain as the main presentation, is considered a predictor of poor postoperative outcomes. The level of compression/herniation is another important factor affecting outcomes. Upper lumbar disc herniation (L1-L2 and L2-L3) has a higher incidence of a poor outcome compared to herniation at lower levels (L3-4, L4-5, and L5-S1). There was no convincing evidence to suggest that surgical technique or type or size of herniation has a significant impact on long-term outcomes. Finally, unemployment and depression were two other factors that predict poor outcomes, and they need to be considered when discussing surgery with patients. We recommend that these factors need to be considered while counseling patients on lumbar decompression surgery for sciatica, as they can predict suboptimal postoperative recovery and outcomes.
